# Properties of Skin Collagen from Southern Catfish (*Silurus meridionalis*) Fed with Raw and Cooked Food

**DOI:** 10.3390/foods13182901

**Published:** 2024-09-13

**Authors:** Qi Zhang, Shufang Hou, Yanmei Liu, Jia Du, Yongkang Jia, Qiushi Yang, Tingting Xu, Yasuaki Takagi, Dapeng Li, Xi Zhang

**Affiliations:** 1 College of Fisheries, National Demonstration Center for Experimental Aquaculture Education, Engineering Research Center of Green Development for Conventional Aquatic Biological Industry in the Yangtze River Economic Belt, Ministry of Education, Hubei Provincial Engineering Laboratory for Pond Aquaculture, Huazhong Agricultural University, Wuhan 430070, China; zhang929@webmail.hazu.edu.cn (Q.Z.); houshufang@webmail.hazu.edu.cn (S.H.); 311621lym@webmail.hzau.edu.cn (Y.L.); 2023308110056@webmail.hazu.edu.cn (J.D.); jiayongkang@webmail.hzau.edu.cn (Y.J.); y357446@webmail.hzau.edu.cn (Q.Y.); 2021308110042@webmail.hzau.edu.cn (T.X.); ldp@mail.hzau.edu.cn (D.L.); 2 Faculty of Fisheries Sciences, Hokkaido University, 3-1-1 Minato-cho, Hakodate 041-8611, Hokkaido, Japan; takagi@fish.hokudai.ac.jp

**Keywords:** southern catfish, type I collagen, biochemical properties, fibril formation, gene expression

## Abstract

The southern catfish (*Silurus meridionalis*) is an economically important carnivorous freshwater fish in China. In this study, we compared the properties of skin collagen from southern catfish fed with raw food (RF) and cooked food (CF). The skin collagen yield in the RF group (8.66 ± 0.11%) was significantly higher than that of the CF group (8.00 ± 0.27%). SDS-PAGE, circular dichroism spectroscopy, and FTIR analyses revealed that the collagen extracted from southern catfish skin in both groups was type I collagen, with a unique triple helix structure and high purity. The thermal denaturation temperature of collagen in the RF group (35.20 ± 0.11 °C) was significantly higher than that of the CF group (34.51 ± 0.25 °C). The DPPH free radical scavenging rates were 68.30 ± 2.41% in the RF collagen and 61.78 ± 3.91% in the CF collagen, which was higher than that found in most fish collagen. Both the RF and CF groups had high ability to form fibrils in vitro. Under the same conditions, the CF group exhibited faster fibril formation and a thicker fibril diameter (*p* < 0.05). In addition, the RF group exhibited significantly higher expression of *col1a1* compared to the CF group. These results indicated that feeding southern catfish raw food contributed to collagen production, and the collagen from these fish may have potential in biomaterial applications.

## 1. Introduction

The southern catfish (*Silurus meridionalis*) is a typical carnivorous freshwater fish, and it is also one of the fastest-growing freshwater fish in China. Southern catfish require feed with a high protein content, especially from animal sources, so cultured fish are mainly fed with living feed. However, fresh feed is costly, limited in quantity, and prone to inducing various diseases and environmental pollution [1]. After a long period of artificial domestication, it became possible to feed this species with compound feeds [2,3]. However, high temperature treatment during feed processing can damage the feed by denaturing protein structures and causing lipid oxidation and loss of amino acids and fatty acids, which ultimately leads to impaired growth performance and health of fish [4]. Therefore, the selection of feeds is particularly important in the culture of southern catfish, as the feed is directly related to the nutritional value and health of the fish, which in turn affects aquaculture efficiency.

To date, studies of southern catfish have focused mainly on culture modes, disease control, and culture efficiency. Little is known about the effects of raw food (RF) and cooked food (CF) on the nutritional structure of this species, and the few existing studies were limited to analyses of the muscle quality and gut microbiological orientation of the fish [4,5]. However, in a study of cuttlefish (*Sepia officianalis*), Domingues et al. [6]. found that specimens fed with high temperature-treated algal shrimp exhibited 13% reduced energy conversion, decreased trypsin activity, and slower growth than those fed raw food. These results suggest that feeding methods can have a significant effect on growth performance, quality, and physiological characteristics of fish. However, the effect of feed type on collagen content has not been reported.

Collagen is one of the most abundant functional proteins in living organisms, accounting for about 30% of total proteins, and it plays an important role in maintaining the structure and function of living organisms [7]. Currently, at least 29 different types of collagens have been identified, of which type I collagen is the most abundant, accounting for >90%. The collagen molecule is a triple helix structure consisting of three polypeptide chains (called alpha chains), the stability of which depends on the hydrogen bonds formed between the polypeptide chains. Proline and hydroxyproline also cause dramatic distortions in the triple helix structure, thus increasing the structural stability of collagen [8].

Collagen fibril formation in vitro is one of the important molecular behavioral features of natural collagen molecules. The in vitro fibril reassembly of collagen is affected by a number of factors, the most important of which are pH and ionic strength [9,10]. The pH affects the rate of assembly of collagen fibrils mainly by changing the charged nature of collagen and its charge size. Ionic strength is due to the role of hydrogen bonding and ionic bonding in the process of collagen fibril reorganization. The effect of ionic strength on collagen fibrils is species-dependent. For example, the rate of fibril formation in sturgeon collagen gradually increased with increasing NaCl concentration. In contrast, the rates of fibril formation of red stingray skin type I collagen and salmon skin type I collagen were suppressed by high NaCl concentrations [11,12]. Understanding the factors that influence collagen fibril self-assembly is necessary for choosing the optimal aggregation conditions when studying different species so that the collagen fibrils can perform optimally.

China is rich in fishery resources and ranks first in the world in terms of fishery production and export. With the export of large quantities of aquatic products, we are also faced with the generation of aquatic waste, including offal, fish skin, fish bones, etc. [13]. Therefore, further processing of aquatic by-products and enhancing the added value of aquatic waste has become an important research topic. Currently, there have been studies to successfully extract trypsin from the intestines of *Coryphaena hippurus*, *Luphiosilurus alexandri,* etc., which is not only easy to extract but also highly profitable [14,15]. Fish skin is an ideal source of collagen as it is a waste product of aquatic products, but its collagen content can be more than 70% of the total protein, which can be used as a natural detergent or applied to many fields such as food, cosmetics, and biomedical industries [16]. Collagen has received increasing attention due to its good biocompatibility, low antigenicity, and high cell adhesion. Especially in tissue engineering, type I collagen is considered one of the best biomaterials for manufacturing scaffolds [17]. In addition, type I collagen has good hygroscopicity, humectancy, emulsification, and emulsion stability, making it suitable for use in the field of skin tissue engineering [18]. Fish-derived collagen is more widely available, less costly, easier to extract, and avoids possible zoonotic diseases and food regulations based on religious beliefs than collagen extracted from mammals [19]. Therefore, fish-derived collagen is expected to be an important alternative source to traditional collagen, and its extraction may be gradually industrialized.

On this basis, the goal of this study was to analyze properties and gene expression levels of collagen from the skin of southern catfish fed either RF or CF and then to compare the results. Our findings can be used to obtain high-quality fish-derived collagen and provide theoretical support for collagen development and application.

## 2. Materials and Methods

### 2.1. Sample Collection and Processing

Southern catfish were cultivated in the indoor recirculating aquaculture experimental system in the College of Aquaculture, Huazhong Agricultural University, China, with a culture period of 6 weeks. During the culture period, southern catfish (6.18 ± 0.52 g, 9.30 ± 0.15 cm) were randomly divided into RF and CF groups, and 10 fish were set up in each group, and each group has 3 replicates. They were separately fed at 10% of their total weight with fresh grass carp (*Ctenopharyngodon idella*) meat or high temperature cooked grass carp meat, respectively, at 07:30 and 19:00.

After completing the cultivation experiment, we randomly selected five fish from each group for analysis. The average body weights and lengths of the fish in the RF group were 27.09 ± 0.89 g and 14.42 ± 0.22 cm, and those of the fish in the CF group were 20.74 ± 0.48 g and 13.58 ± 0.25 cm. We anesthetized the fish using MS-222 (140 mg/L), collected skin samples, and temporarily stored them in liquid nitrogen. After all sampling was complete, we stored the samples at −20 °C until used in subsequent experiments.

### 2.2. Isolation and Purification of Collagen

We used the acid-enzyme method to extract collagen from the southern catfish skin samples. The fish skin was thawed in a thermostat at 4 °C and then weighed to obtain the initial weight of fresh fish skin (m_0_). The fresh fish skin was placed in a freeze dryer for 24 h to obtain the dry weight of the skin (m_1_). After cutting the skin into small pieces (0.5 cm × 0.5 cm), we treated the samples with 0.1 M NaOH (Sinopharm Chemical Reagent Co., Ltd., Shanghai, China) for 24 h (*w*/*v* = 1:20) to remove the pigments and heteroproteins. After washing away the NaOH, the fish skin was treated with 99.5% anhydrous ethanol for 24 h (*w*/*v* = 1:30) to remove fat, and then the sample was washed with distilled water. The pretreated fish skin was treated with 0.5 M acetic acid (Sinopharm Chemical Reagent Co., Ltd., Shanghai, China) containing 0.2% porcine pepsin (EC 3.4.23.1, 1:10,000; Sigma-Aldrich, St. Louis, MO, USA) for 72 h (*w*/*v* = 1:30) to obtain the collagen crude extract. We centrifuged the crude extract at 8000× *g* for 20 min and then recovered the supernatant. We added NaCl to the supernatant to bring the concentration of the solution to 1 M for salting out and then centrifuged the mixture at 8000× *g* for 40 min. The precipitate was dissolved in 0.5 M acetic acid (*w*/*v* = 1:30). The salting-out process was repeated three times to obtain the refined collagen extract. All experimental procedures were carried out at 4 °C. Each extract was dialyzed in 50 times the volume of distilled water for 2 days and then placed in a freeze dryer to obtain solid collagen, which was weighed to obtain the final weight (m_2_). The extraction rate (y) was calculated using the formula:

Y_wet_ = m_2_/m_0_ × 100%.

Y_dry_ = m_2_/m_1_ × 100%.

### 2.3. Sodium Dodecyl Sulfate Polyacrylamide Gel Electrophoresis (SDS-PAGE)

An aliquot of the freeze-dried collagen from each sample was dissolved in HCl solution (pH = 3.0, 3 mg/mL), mixed with the sample buffer (5×) (Guangzhou Jefass Biotechnology Co., Ltd., Guangzhou, China) in a ratio of 4:1, and heated at 95 °C for 15 min. Electrophoresis was carried out on a 5% concentrate gel and 7.5% separator gel. After electrophoresis, the samples were stained with Coomassie Brilliant Blue R250 solution (Thermo Fisher Scientific, Waltham, MA, USA) staining solution for 30 min, then decolorized with Kaomas Brilliant Blue decolorizing solution, and the results of the bands were observed. The relative band density of the α1 chain and α2 chain were analyzed using Image J software (Version 1.8.0).

### 2.4. Circular Dichroism (CD) Measurement

Another portion of the lyophilized collagen from each sample was dissolved in HCl solution (pH = 3.0, 1 mg/mL) and placed in a 0.1 cm optical diameter quartz cuvette. At 25 °C, the CD spectra were measured at a scanning speed of 50 nm/min and 0.1 nm intervals at a wavelength range of 190 to 250 nm. The 221 nm CD spectrum was measured as the temperature increased from 25 to 45 °C at 1 °C/min. The temperature at the point of maximum slope of CD change was determined as the denaturation temperature of collagen.

### 2.5. Attenuated Total Reflectance Fourier Transform Infrared (ATF-FTIR) Spectroscopy

ATR-FTIR spectroscopy analysis of portions of the lyophilized collagen samples was conducted using a Fourier Transform Infrared Spectrometer (Nicolet IS50; Thermo Fisher Scientific). The scanning range was 600 to 4000 cm^−1^ with a resolution of 4 cm^−1^, and the number of scans was 16 for both background and sample after atmospheric background deduction.

### 2.6. Antioxidant Activity

Lyophilized collagen samples were dissolved in HCl solution (pH = 3.0) to reach a final concentration of 3 mg/mL. We then measured the antioxidant capacity of collagen from southern catfish skin using the 2,2-diphenyl-1-picrylhydrazyl (DPPH) free radical scavenging capacity kit (Nanjing Construction Institute of Biological Engineering Co., Ltd., Nanjing, China).

### 2.7. Collagen Fibril Formation In Vitro

Aliquots of the lyophilized collagen from each sample were dissolved in HCl solution (pH = 3.0) to obtain a 3 mg/mL solution. After the samples were completely dissolved, the collagen solutions were mixed with 0.1 M phosphate buffered saline (PBS) at pH 7.0 and different salinities (NaCl = 0, 140, or 280 mM) (sample solution ratio = 1:2). Subsequently, a small amount of the mixed solution was placed in an ultraviolet spectrophotometer (Shanghai Meitu Instrument Co., Ltd., Shanghai, China), and the absorbance values were recorded every 3 min. The rate of collagen formation at different NaCl concentrations was compared based on the changes in absorbance value at 321 nm.

### 2.8. Determination of Collagen Fibrils Formed In Vitro

Twenty-four hours after collagen fibrils were formed, each mixture was centrifuged at 12,000× *g* for 20 min using a micro cryo-centrifuge (model D3024R; Scilogex, Rocky Hill, CT, USA), and the precipitate was collected. At room temperature, 1 mL of electron microscope fixative containing 2.5% glutaraldehyde (No. G1102; Wuhan Xavier Biotechnology Co., Ltd., Wuhan, China) was added to the centrifuge tube, and the sample was fixed for 4 h. We washed each precipitate with PBS buffer and then sequentially treated it with ethanol solutions with volume fractions of 30%, 50%, 70%, and 100%. After gradient dehydration, each collagen fibril precipitate was treated with 1 mL tert-butanol, and the treatment was repeated twice. After removing the tert-butanol solution, the centrifuge tubes were placed in a freezer overnight at −80 °C, and then they were dried in a vacuum-drying freezer.

For analysis, we tore a small piece of fibrous film from the collagen fibrils using pointed tweezers, placed it on conductive adhesive, and then plated the surface using an ion sputtering apparatus (MC1000; Hitachi, Tokyo, Japan). The plated collagen fibrils were observed using a field emission scanning electron microscope (model SU8010; Tianmei Scientific Instruments Co., Ltd., Shanghai, China) at 20,000 times magnification. The diameters of 150 of the reassembled collagen fibrils from each sample were manually calculated using Image-Pro Plus software (Version 6.0, Media Cybernetics, Silver Spring, MD, USA), and the average value was calculated.

### 2.9. Analysis of the Expression of Genes Related to Collagen Synthesis

#### 2.9.1. Total RNA Extraction, Purification, and Complementary DNA (cDNA) Synthesis

Total RNA was extracted from southern catfish skin using TRIzol^®^ RNA Extraction Solution (Thermo Fisher Science) following the manufacturer’s instructions. Fish skin tissue was cut into small pieces and placed into 1.5 mL enzyme-free centrifuge tubes. To determine the RNA integrity, samples were separated on 0.8% nondenaturing agarose gel. The total RNA concentration was determined using a micro-volume spectrophotometer (NanoDrop™ 2000; Thermo Fisher Scientific Inc.). Subsequently, the concentration was adjusted to 200 ng/μL using nuclease-free water for cDNA synthesis. The cDNA was synthesized from the RNA using a reverse transcription kit (Hifair^®^ III 1st Strand cDNA Synthesis SuperMix for qPCR; YEASEN, Shanghai, China). The reaction was performed in a T100 PCR thermocycler (Bio-Rad Laboratories; Hercules, CA, USA) with incubation at 25 °C for 5 min, 55 °C for 15 min, and 98 °C for 5 min.

#### 2.9.2. Real-Time PCR (RT-PCR) Expression Assay

The cDNA obtained above was diluted 5-fold and used as a template for RT-PCR. Table 1 lists the primers used in the analysis. The 20 μL reaction system was prepared in a 96-well plate according to the instructions of the Hieff^®^ qPCR SYBR^®^ Green Master Mix (Low Rox Plus) kit (YEASEN, Shanghai, China). After sufficient mixing, the amplification program was performed. The relative expression of target genes was analyzed and calculated using the 2^−∆∆ct^ method.

### 2.10. Statistical Analysis

Data are presented as the mean ± standard error (SE). Statistical analyses were performed using SPSS Base 25 statistical software (IBM, Armonk, NY, USA). After testing fornormal distribution and homogeneity of variance of the trial data, comparison among data within groups was conducted using one-way analysis of variance followed by Dun-can’s tests. *p* < 0.05 was considered to be statistically significant. In all graphs, SC means collagen, RF is collagen in the raw food group, and CF is collagen in the cooked food group.

## 3. Results and Discussion

### 3.1. Yields of Collagen

**Figure 1:**
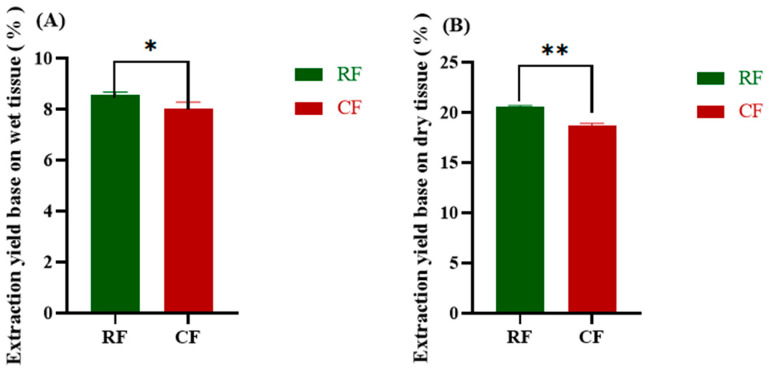
(**A**) Wet weight extraction rates of collagen from southern catfish skin under raw (RF) and cooked (CF) conditions. (**B**) Dry weight extraction rates of collagen from southern catfish skin under raw (RF) and cooked (CF) conditions. * denotes a significant difference between RF and CF (*p* < 0.05). ** indicates highly significant differences (*p* < 0.01).

The yields of collagen from fish skin in the RF and CF groups are shown in Figure 1. The extraction rate based on wet tissue was 8.66 ± 0.11% in the RF group and 8.00 ± 0.27% in the CF group. The extraction rate based on dry weight was 20.53 ± 0.03% in the RF group and 18.68 ± 0.23% in the CF group. The RF group had a higher yield than that of the CF group in both cases. The wet weight extraction rate of southern catfish skin collagen was slightly lower than that of grass carp skin collagen (10.61 ± 0.67%), snapper skin collagen (13%), and tilapia (*Oreochromis niloticus*) skin collagen (27.2%), but higher than that of bigeye snapper skin (7.5%) [20,21,22,23]. The dry weight extraction rate was similar to that of black drum fish (18.1%), but lower than that of ocellate puffer fish skin (55.4%) and largefin longbarbel catfish skin (44.8%) [24,25].

The yield of collagen is related to factors such as the species and size of fish, the extraction method, and the method of yield calculation. The relatively low collagen extraction yield in this study may be related to the small size of the fish and its thin skin. In addition, the solubility and yield of collagen are also related to interchain cross-links in its terminal peptide region. These crosslinks undergo vibrational cleavage in the presence of acids or enzymes, leading to an increase in collagen yield [26]. Ultrasound-assisted enzymatic extraction of skin collagen from Alaskan pollock (*Theragra chalcogramma*) gave a yield of 65.30%, and skipjack tuna (*Katsuwonus pelamis*) gave a final stable yield of 38.11% of skin collagen [27,28]. These studies suggest that the use of novel collagen extraction methods in combination with traditional extraction methods can greatly increase collagen yield and is an important way to industrialize collagen. However, the effect of new applications such as ultrasound on collagen biological activity properties, including fibril formation, is not known. In the future, it is possible to optimize the extraction method to increase the collagen extraction rate without affecting the collagen properties of southern catfish.

### 3.2. SDS-PAGE

**Figure 2:**
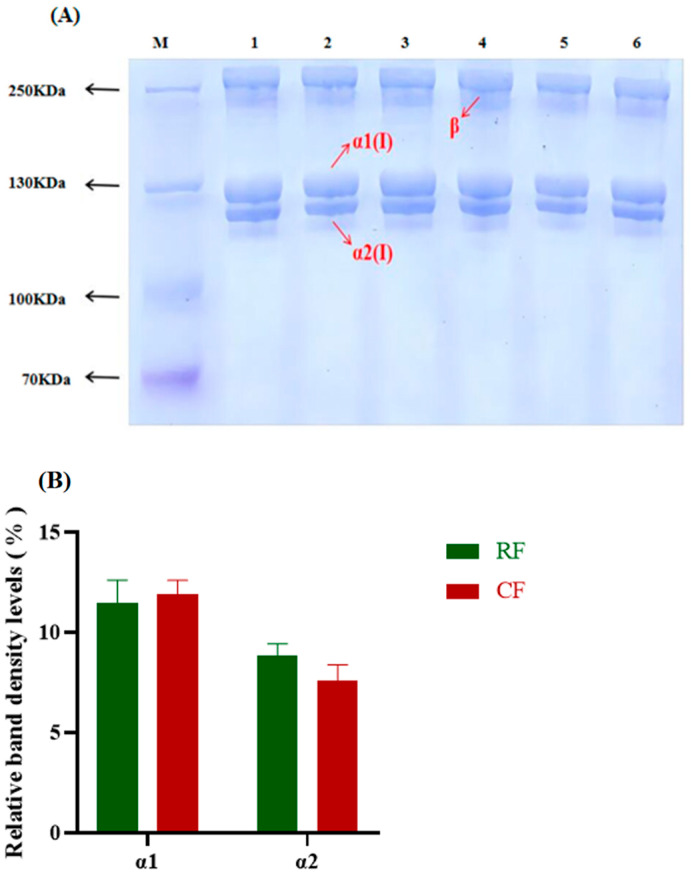
(**A**) SDS-PAGE results of skin collagen from southern catfish skin under raw food (RF) and cooked food (CF) feeding. M: Marker; 1–3: RF feeding; 4–6: CF feeding. (**B**) Relative band density levels of α1 and α2 chains.

The SDS-PAGE results showed that the collagen of southern catfish skin mainly consisted of two α-bands (~130 kDa and ~120 kDa) and a β-band with a molecular weight of more than 250 kDa, with no additional heterogeneous bands (Figure 2A). According to the molecular weight and its morphology, the about 130 kDa band was the α1 chain, and the about 120 kDa band was the α2 chain. This is consistent with the electrophoretic properties of type I collagen, and it was consistent with the structure of the electropherogram of collagen purified from southern catfish skin in a previous study [29]. These results indicate that the collagen from southern catfish skin belongs to type I collagen. The extracted collagen was free from impurities and had high purity. The relative levels of the α1 chain and α2 chain were similar between the RF and CF groups (Figure 2B). In this study, there was no significant difference in the molecular weights and relative levels of the α1 and α2 chains of southern catfish skin collagen between the RF and CF groups, but both of them had complete triple-helical structures, which had the potential value of being applied as a source of collagen or gelatin. In addition, they have the advantage of being extractable from the epidermis of aquatic by-products. Although most of the resources are currently derived from mammals, increasing attention is being paid to non-mammalian resources, such as fish, suggesting the potential for wider utilization in the future [30].

### 3.3. ATR-FTIR

**Figure 3:**
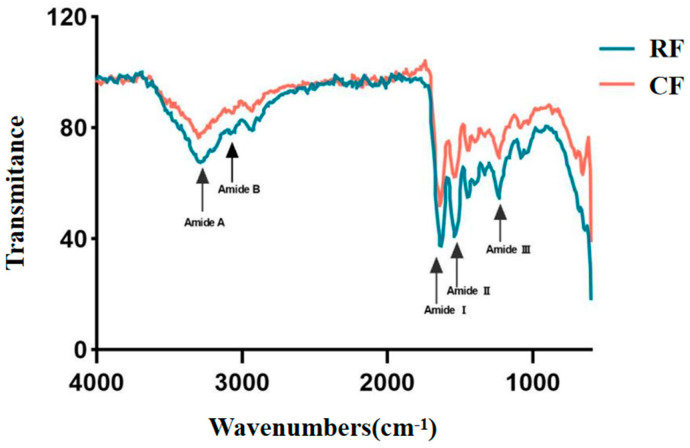
Infrared spectra of skin collagen from southern catfish skin under raw food (RF) and cooked food (CF) feeding.

ATR-FTIR is used to analyze the molecular structure by detecting changes in the chemical bonds in the molecule. Fish skin collagen from RF and CF groups had similar infrared spectra, but subtle differences in the collagen could be detected (Figure 3). The IR spectra contained five characteristic peaks of type I collagen, amide A and B bands, and amide I, II, and III bands. The presence of absorption peaks in the amide I, II, and III bands indicates that the triple helical structure of type I collagen we extracted from the skin of southern catfish is intact, which is similar to the results of infrared spectroscopy reported for collagen from grass carp [20].

The absorption peaks in the amide A band are caused by N-H stretching, and the free N-H stretching vibration range is usually 3400–3440 cm^−1^ [31]. In our experiments, the stretching vibration of the amide A band of southern catfish skin collagen in the RF group appeared at 3310 cm^−1^, while that of the amide A band in the CF group appeared at 3300 cm^−1^, and both values were shifted to shorter wavelengths compared with the free N-H stretching vibration range, a phenomenon known as the blue shift [32]. A blueshift in FTIR spectra can occur due to the reduction of hydrogen bonding, which alters internal vibration and rotation of the molecules and causes absorption peaks to shift to shorter wavelengths. The greater blueshift of the CF group compared to the RF group suggested that fewer hydrogen bonds are present in the skin collagen under CF conditions. The amide I and amide II bands appeared at 1640 cm^−1^ and 1544.4 cm^−1^ in the RF group and 1638 cm^−1^ and 1540.7 cm^−1^ in the CF group, respectively. The absorption waves of the amide I and II bands were higher in the RF group than in the CF group, which suggests that the degree of ordering and cross-linking of the fish skin collagen was higher in the RF group than in the CF group [33].

### 3.4. Thermal Stability

**Figure 4:**
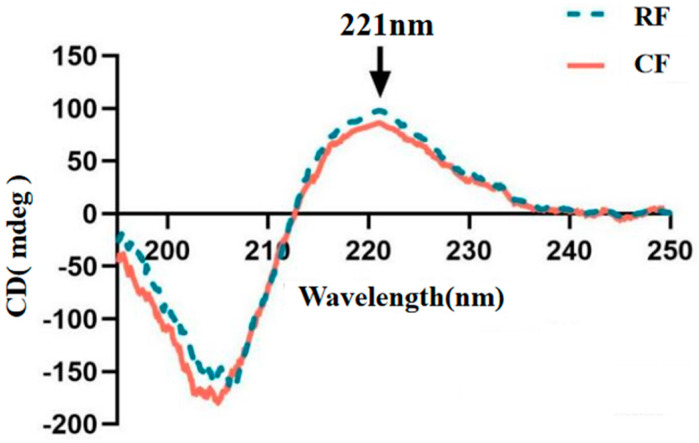
Collagen CD spectra of southern catfish skin under raw food (RF) and cooked food (CF) feeding.

Figure 4 shows the results of CD spectra analysis of southern catfish skin collagen. CD spectra provide information on the secondary structure of collagen [34]. All collagens showed a rotatory maximum at 221 nm and a crossover point (zero rotation) at about 212 nm, which are typical characteristics of the collagen triple helical conformation [35,36]. The single negative absorption peak indicated a predominantly β-folding, which is consistent with the typical characteristics of type I collagens [37], which have a complete triple helical structure. These peaks are important markers for determining collagen type. The maximum positive UV absorption peak of southern catfish skin collagen was 221 nm in both RF and CF groups, indicating a standard collagen triple helix structure with biological activity in both groups, which were suitable for denaturation temperature measurements and fibril formation experiments, yet there was no significant difference between the CF and RF groups. Previous studies reported a maximum UV absorption peak of 235 nm for collagen extracted from pufferfish (*Lagocephalus inermis*) skin and 220 nm for jellyfish (*Acromitus hardenbergi*) [38,39]. These varying results suggested that collagen raw materials and extraction methods affected the UV absorption peaks of collagen, which may be related to the exposure of the chromogenic modes in the collagen peptide chain [40].

**Figure 5:**
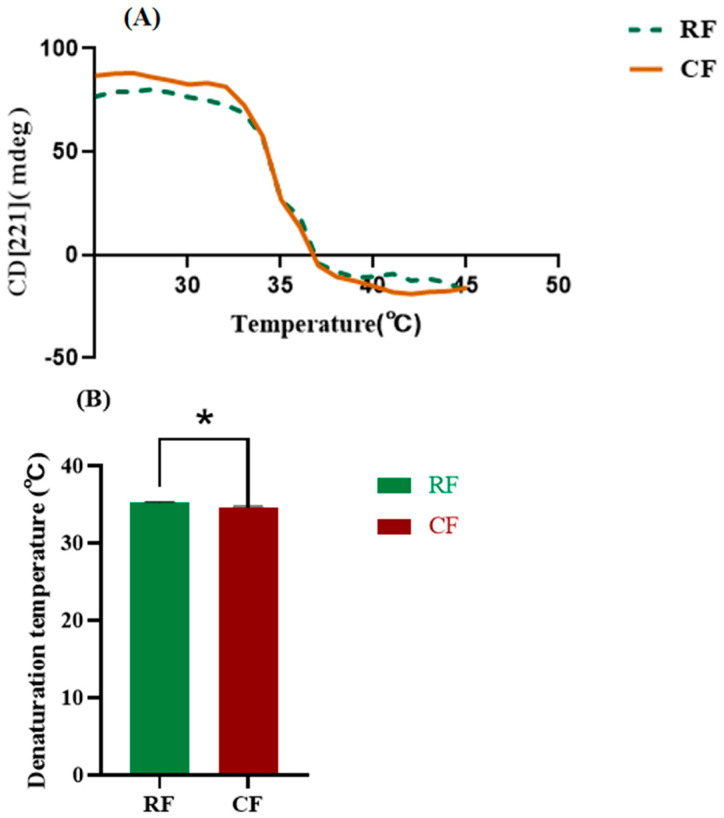
(**A**) Effects of temperature on collagen CD of catfish skin at 221 nm; (**B**) Collagen denaturation temperature of southern catfish skin. * Indicates significant differences between RF and CF (*p* < 0.05).

The thermal denaturation temperature of collagen is an important index for evaluating its thermal stability. When heated, the triple helix structure of collagen begins to unravel to form a single chain due to the breaking of hydrogen bonds. The thermal denaturation temperature is the temperature at which the rate of unraveling is 50% [41]. In our study, the thermal denaturation temperature of collagen in the RF group (35.20 ± 0.11 °C) was significantly higher than that in the CF group (34.51 ± 0.25 °C) (*p* < 0.05), which may be related to the high content of imino groups (proline and hydroxyproline) and hydrogen bonding in the southern catfish fed RF [42]. The FTIR analysis revealed that fish skin collagen under the RF feeding condition had more hydrogen bonding content, a higher cross-linking ratio, and a more stable triple helical structure, which was consistent with the result of higher denaturation temperature and a better thermal stability capacity in RF collagen (Figure 5). The thermal stability of collagen is also related to the solution environment, raw material source, season, and living environment, and usually the denaturation temperature of collagen from cold-water fish is lower [43]. We found that southern catfish skin collagen was thermally more stable than skin collagen from tilapia (denaturation temperature of 31 °C), Atlantic cod (*Gadus morhua*) (denaturation temperature of 29.6 °C), and sturgeon (*Acipenser schrenckii*) (denaturation temperature of 28.5 °C) [22,44,45]. However, the denaturation temperature of fish-derived collagen is usually lower than that of mammalian collagen, which is a substantial limiting factor for collagen applications [46]. Currently, collagen thermal stability can be improved by modification. Tian et al. [47] reported that the denaturation temperature of collagen could be increased by 27 °C when it was cross-linked with glutaraldehyde, which greatly enhanced the thermal stabilization ability of collagen and provided a possibility for fish-derived collagen to replace mammalian collagen in biomedical and other fields.

### 3.5. DPPH Free Radical Scavenging Capacity

**Figure 6:**
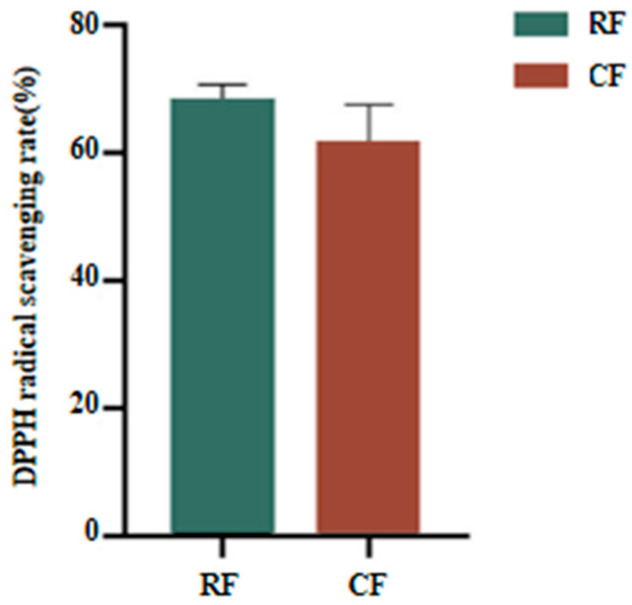
DPPH radical scavenging rate of skin collagen from southern catfish skin under raw food (RF) and cooked food (CF) feeding.

Superior antioxidant properties are one of the main reasons why collagen is widely used in cosmetics, functional food ingredients, and additives [38]. The DPPH free radical scavenging rate of southern catfish skin collagen was higher in the RF group (68.30 ± 2.41%) than that of the CF group (61.78 ± 3.91%), but the difference between the two was not statistically significant (Figure 6). The antioxidant activity of collagen from the skin of southern catfish was higher than that of crimson snapper collagen (39.57 ± 0.99%), silver pomfret collagen (40.89 ± 0.22) and Malabar sole (31.4 ± 0.96%). [48]. The collagen extracted in this study showed excellent in vitro antioxidant capacity compared to the antioxidant properties of commercially available collagen (DPPH free radical scavenging rate of about 30–40%) [38]. The subtle difference in antioxidant capacity of collagen between RF and CF groups may be related to the amino acid composition, structure, hydrophobicity, and molecular weight of collagen [49]. Chen et al. [50] reported that the excellent antioxidant properties of collagen could be helpful in the development of wound dressings that could prevent oxidative stress induced by excess free oxygen at the wound site, thereby promoting wound healing.

### 3.6. Collagen In Vitro Fibril Formation

**Figure 7:**
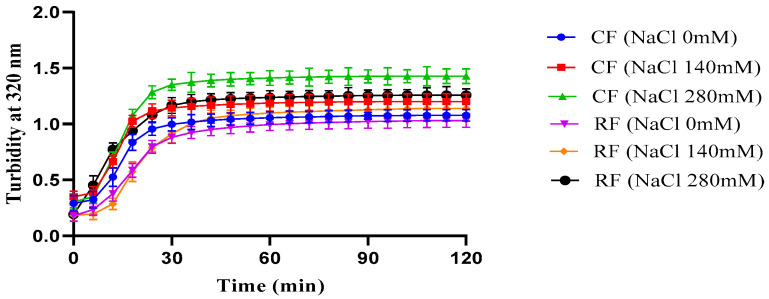
Effect of salinity on collagen fibril formation rate of skin collagen from southern catfish skin under raw food (RF) and cooked food (CF) feeding.

In vitro self-assembly capacity is one of the important molecular behavioral features of natural collagen molecules. In this study, collagen self-assembly in vitro was investigated by the turbidimetric method. The slope of the turbidity curve reflects the rate of lateral aggregation, while the final turbidity reflects the fibril diameter [51]. Usually, the turbidity curve of collagen fibrils is typically S-shaped and is divided into three periods: the hysteresis period, the growth period, and the stability period. Among them, the hysteresis period is the stage of collagen fibril nucleation, with no absorbance change; the absorbance increases rapidly during the growth period, and collagen self-assembles into fibrils; and the maximum absorbance value is reached during the stabilization period, and the collagen fibril mesh structure is formed [52]. In this study, the experimental results are shown in Figure 7, and the turbidity curves do not show any significant lag period, which indicates that collagen nucleation in southern catfish is extremely fast and can be rapidly nucleated in a short period of time. In contrast, in the porcine collagen fibrillogenesis study, the collagen turbidity curve had an obvious nucleation period, and the assembly rate during the growth period was significantly smaller than that of fish-derived collagen, which showed a better fibrillogenic capacity of fish compared with mammalian collagen [53,54]. This result is consistent with the inference from SDS-PAGE that low molecular weight components contribute to promoting fiber formation.

The slope of the turbidity curve reflects the rate of fibril formation, while the final turbidity reflects the fibril diameter to some extent [51]. In the present study, the growth period of collagen fibril formation appeared to be faster, with higher final turbidity and coarser fibril diameters in CF than in RF under the same salinity conditions. Different NaCl concentrations lead to different ionic strengths of the solution, which affect the assembly process of collagen molecules [54]. The slope of collagen fibril formation was minimum at a salinity of 0 mM and maximum at a salinity of 280 mM. This suggests that increasing NaCl concentration within a certain range promotes collagen fibril formation of both RF and CF groups. We speculate that the possible reason is that increased NaCl concentration results in enhancing ionic interactions, which may promote more effective fibril assembly and cross-linking. The result that increased salinity accelerates collagen fibril formation in southern catfish is similar to our previous findings in sturgeon and grass carp collagen [20,44,54]. The general principles of collagen fibril formation are accepted, but the molecular mechanism of the assembly process is less known.

### 3.7. Morphology of Collagen Fibrils Formed In Vitro

**Figure 8:**
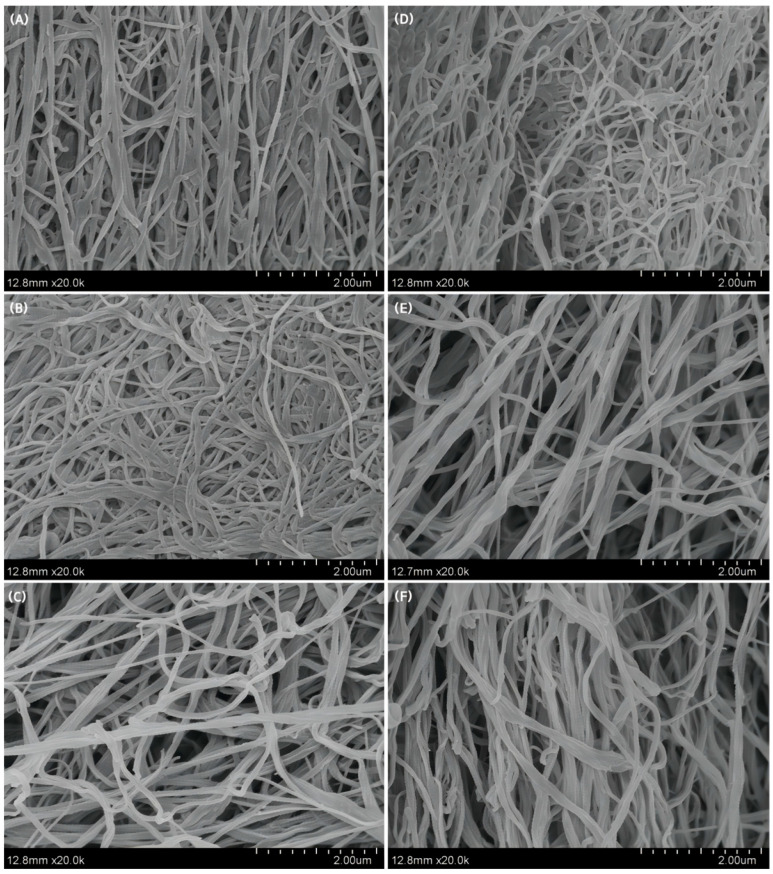
Scanning electron microscope images of collagen fibrils obtained from the skin of southern catfish. (**A**–**C**): raw food (RF) feeding, NaCl concentration was 0 mM, 140 mM, and 280 mM, respectively; (**D**–**F**): cooked food (CF) feeding, NaCl concentration was 0 mM, 140 mM, and 280 mM, respectively.

**Figure 9:**
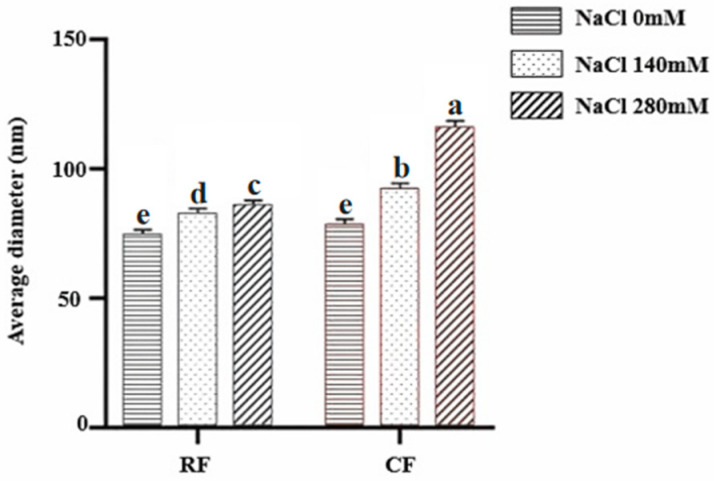
Effect of salinity on the diameter of skin collagen fibrils from southern catfish skin under raw food (RF) and cooked food (CF) feeding (*n* = 150, unit in nm). Different lowercase letters indicate differences among different salinities and groups (*p* < 0.05).

Figure 8 shows the morphology of the reconstituted collagen fibrils of CF and RF observed by scanning electron microscopy. The reconstituted collagen fibrils showed clear fibrous aggregated structures at 0, 140, and 280 mM salinity. The average diameter of self-assembled collagen fibrils was the smallest at 0 mM salinity and the largest at 280 mM salinity in both RF and CF groups (Figure 9), and the effect was more pronounced in the CF group.

The effects of NaCl concentration on collagen-forming fibrils in fish skin from both RF and CF groups were basically the same; increasing the NaCl concentration resulted in an increase in the diameter of collagen fibrils, which is consistent with the results of the collagen fibril formation process. This result suggests that the overall trend of the effect of NaCl concentration on collagen fibril formation in both groups is consistent, but the impact is more pronounced in CF groups. At the same NaCl concentration, the collagen fibril formation rate in the CF group was faster than that in the RF group, and the final collagen fibrils formed were thicker in diameter; thus, CF groups had a better fibril-forming ability than RF groups. In addition, the aforementioned ATR-FTIR results showed that the collagen of southern catfish skin under CF modes had less hydrogen bonding, which may also allow collagen to have better nucleation and assembly capacity during fibril formation. Our findings suggest that southern catfish collagen has a rapid fibrillogenic capacity and a very distinct network structure. These findings highlight the importance of studying the fibrillar behavior of collagen for collagen development and application, especially in the fields of tissue engineering and biomaterials.

### 3.8. Collagen Synthesis-Related Gene Expression

**Figure 10:**
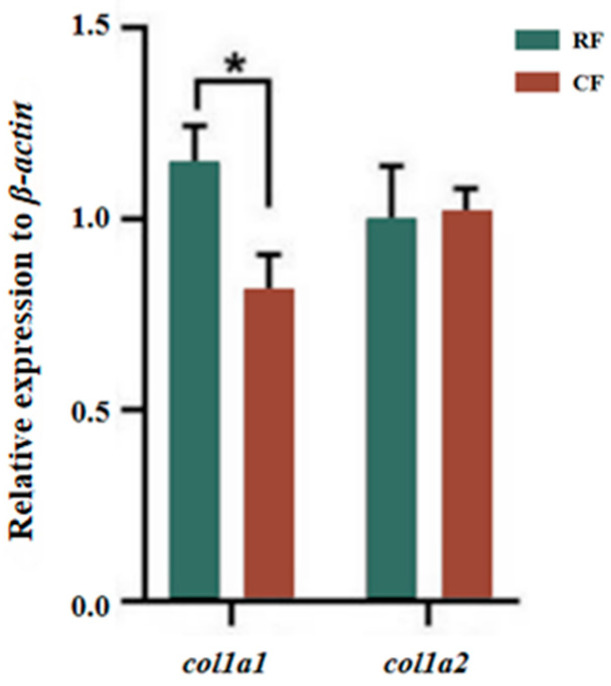
Effects of different feeding modes on gene expression of type I collagen in southern catfish skin. RF, raw food feeding; CF, cooked food feeding. * Indicates significant differences among different salinities (*p* < 0.05).

The mRNA expression levels of the *col1a1* genes in the fish skin of the RF group were significantly higher than those of the CF group (*p* < 0.05), but the mRNA expression level of the *col1a2* gene did not differ significantly between the two groups (Figure 10). The synthesis of collagen peptide chains is mainly encoded by the *col1a1* and *col1a2* genes. After these genes are expressed, the corresponding polypeptide chains α1 and α2 form, and two α1 chains and one α2 chain together form type I collagen with a three-stranded helical structure [55]. Therefore, the mechanism responsible for enhanced collagen content in the RF group may be through inducing the up regulation of *col1a1* and *col1a2* expression, thereby increasing the production of peptide chains α1 and α2. The higher expression levels of *col1a1* mRNA in the RF group could promote the synthesis of collagen, which is consistent with the collagen yield results. However, the stimulation of *col1a1* expression alone in the RF group suggests that the dietary difference induced a difference in the ratio of peptide chains α1 and α2. Such compositional changes in collagen peptide chains may relate to the differences in the nature of collagen molecules reported above. Along with the expression studies on *col1a3*, which could not be studied in the present study, the effects of the diet on the composition of collagen peptide chains should be studied in the future.

## 4. Conclusions

In this study, we investigated the properties and fibril-forming ability of skin collagen from southern catfish fed with raw and cooked food and assessed the synthesis of collagen at the gene level. Our results suggested that southern catfish skin collagen was type I collagen with a unique triple helix structure. The relative expression of *col1a1* was higher in the RF group than in the CF group, which resulted in higher collagen synthesis and higher collagen yield as well as certain advantages in denaturation temperature and antioxidant capacity. Collagen in both groups showed good fibril-forming ability, and the self-assembling behavior of collagen could be regulated by NaCl concentration. The CF group had a faster assembly rate of collagen and higher fibril-forming ability compared with the RF group, illustrating that feeding fish-cooked food might produce collagen with higher potential applications in biomaterials. In conclusion, the collagen of southern catfish skin had high thermal stability, antioxidant effects, and fibril-forming abilities, which suggested the broad application potential of this material in cosmetics, biomedicine, and tissue engineering. Additionally, RF and CF had certain effects on collagen properties. Our results provided an important basis for the development and utilization of by-products from southern catfish. Furthermore, this study is the first to explore the effects of different processing methods of food on southern catfish collagen, and the results suggest that raw food is more beneficial for collagen production and some properties. This provides essential data for the future development of food and feed products for southern catfish.

## Figures and Tables

**Table 1:** Primers sequence of real-time PCR.

Gene	Primer Sequence (5′-3′)
*β-actin-F*	GATCCGGTATGTGCAAGGCT
*β-actin-R*	TGCCAGATCTTCTCCATATCA
*col1a1-F*	AGCTTACCTTCTTGCGCCTT
*col1a1-R*	GACGCTGTATGTGAAACGGC
*col1a2-F*	ACAAGGAGTCTGCATGTCGG
*col1a2-R*	TATCTCCCCTTGGTCCCGAT

## Data Availability

The original contributions presented in the study are included in the article, further inquiries can be directed to the corresponding author.
